# *rac*-2-{3-[1-(Acet­yloxy)eth­yl]-2,2-di­methyl­cyclobut­yl}acetic acid

**DOI:** 10.1107/S2414314624011441

**Published:** 2024-12-03

**Authors:** Dieter Schollmeyer, Paul Jirsch, Heiner Detert

**Affiliations:** a University of Mainz, Department of Chemistry, Duesbergweg 10-14, 55099 Mainz, Germany; Goethe-Universität Frankfurt, Germany

**Keywords:** crystal structure, cyclo­butane, strain

## Abstract

The title compound, C_12_H_20_O_4_, was prepared from α-pinene in three steps. The ester and acid moieties are *cis* on the slightly folded cyclo­butane ring. In the crystal, carb­oxy­lic acid bound dimers form layers parallel to (202).

## Abstract 2

The title compound was prepared from α-pinene in three steps. The ester and acid moieties are *cis* on the slightly folded cyclo­butane ring. In the crystal, carb­oxy­lic acid bound dimers form layers parallel to (202).

## Graphical abstract

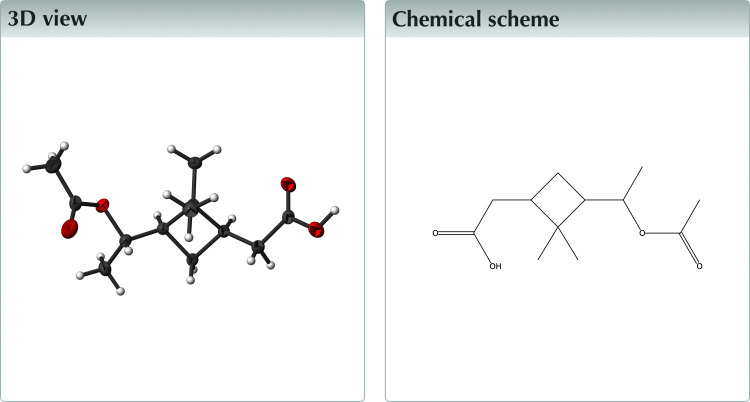

## Structure description

**Figure 1:**
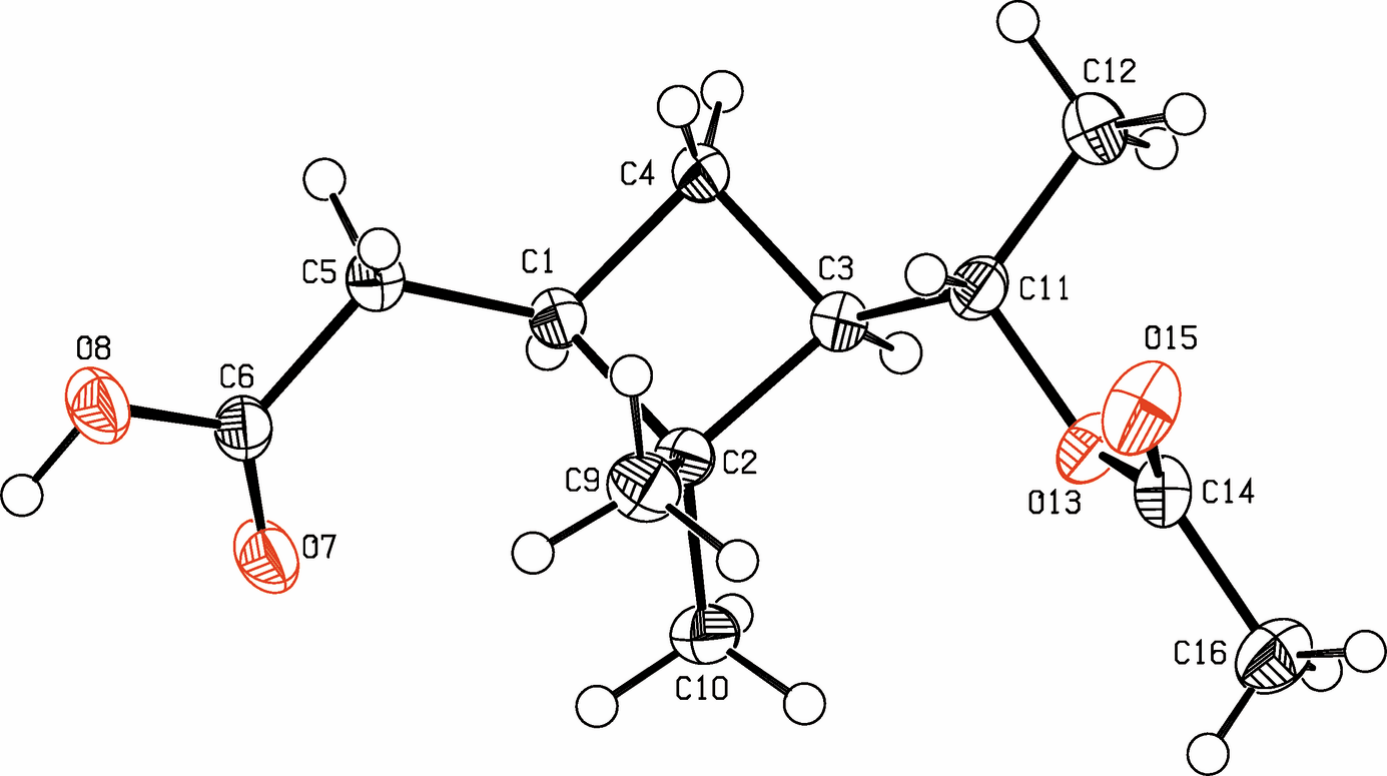
View of the title compound. Displacement ellipsoids are drawn at the 50% probability level.

**Figure 2:**
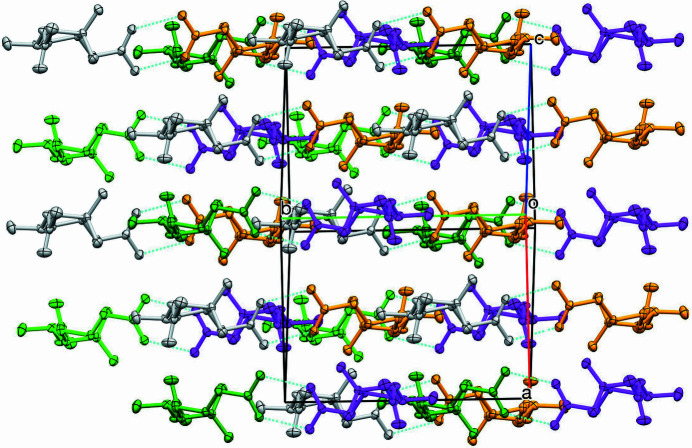
Part of the packing diagram. Hydrogen bonds are drawn with dashed lines. View along the [101] direction. The color of the mol­ecules corresponds to the generating symmetry operator.

As part of a project on strained carbocycles (Detert & Schollmeyer, 2017 ▸; Herges *et al.*, 2005 ▸), the title compound, C_12_H_20_O_4_ (Fig. 1 ▸), was prepared from racemic α-pinene by permanganate oxidation, borohydride reduction of the pinonic acid to pinolic acid and acetyl­ation. The compound crystallizes in the monoclinic space group *C*2/*c* with the asymmetric unit containing eight mol­ecules. Two enanti­omeric mol­ecules are connected *via* two hydrogen bridges of the carb­oxy­lic acids, forming centrosymmetric dimers. The distance between the oxygen atoms forming the hydrogen bond is 2.6547 (13) Å. These dimers are arranged in layers parallel to the (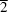
02) plane (Table 1 ▸, Fig. 2 ▸). The central cyclo­butane ring is folded in a butterfly-like manner: the planes defined by C1,C2,C4 and by C2, C3, C4 subtend an angle of 24.61 (12)°, which is due to the bulky methyl groups at C2. However, it is significantly smaller than the ideal angle of 35° (Bucourt, 1974 ▸). The acetic acid substituent on C1 and the acet­oxy­ethyl on C3 are *cis* and on the open side of the folded cyclo­butane. The geminal methyl groups on C2 open an angle of 110.39 (10)° and provoke an elongation of the cyclo­butane bond lengths *e.g.* C1—C2 = 1.5697 (15) Å *versus* C1—C4 = 1.5467 (15) Å. A deviation of only 0.0193 (10) Å for O8 destroys the otherwise perfect planarity of the acetic acid unit O7,O8,C5,C6.

## Synthesis and crystallization

The title compound was prepared from α-pinene by phase-transfer-catalyzed oxidation with permanganate according to Hünig *et al.* (1979 ▸) (43% yield) followed by reduction with sodium borohydride according to Fernández *et al.* (2001 ▸) (94% yield). The resulting diastereomeric mixture of pinolic acids (2.00 g) was dissolved in benzene (5 ml), acetic acid (2.58 g) and toluene­sulfonic acid (0.47 g) were added. The mixture was refluxed for 3.5 h and water was separated using a Dean–Stark trap. The mixture was washed with water, the aqueous phase extracted with toluene and the combined organic layers were dried and the solvents removed *in vacuo*. The residue thus obtained was dissolved in heptane (5 ml), treated with active charcoal and filtered. Upon cooling, the mixture separated into two phases, the lower layer was dissolved in heptane (15 ml) and upon cooling for 3 days. The precipitated solid was recrystallized from heptane to yield 0.22 g (9%) of colorless crystals with m.p. = 360–362 K. Hergueta *et al.* (2003 ▸) report a melting point of the enanti­opure compound of 258-258 K. Their NMR data correspond well with the results from the racemate, except a general deep-field shift of all H-NMR signals and a high-field shift of *ca* 0.25 p.p.m. in C-NMR. The numbering of H- and C-signals follows IUPAC nomenclature. ^1^H-NMR (300 MHz, CDCl_3_): δ = 4.77 (*dq*, *J* = 10.2, 6.2 Hz, 1H, 1′′-H), 2.41–2.15 (*m*, 3H, 2-H, 1′-H), 2.14–1.93 (*m*, 2H, 3′-H, 4′-H), 2.00 (*s*, 3H, 4′′-H), 1.30–1.16 (*m*, 1H, 4′-H), 1.08 (*s*, 3H, 5′′-H), 1.06 (*d*, *J* = 6.2 Hz, 3H, 2′′-H), 0.88 (*s*, 3H, 6′′-H). ^13^C-NMR (101 MHz, CDCl_3_): δ = 179.3 (C-1), 170.7 (C-3′′), 71.9 (C-1′′), 47.1 (C-3′), 40.0 (C-2′), 37.9 (C-1′), 35.0 (C-2), 30.5 (C-5′′), 26.5 (C-4′), 21.6 (C-4′′), 17.7 (C-2′′), 16.9 (C-6′′).

## Refinement

Crystal data, data collection and structure refinement details are summarized in Table 2 ▸.

## Supplementary Material

Crystal structure: contains datablock(s) I, global. DOI: 10.1107/S2414314624011441/bt4161sup1.cif

Structure factors: contains datablock(s) I. DOI: 10.1107/S2414314624011441/bt4161Isup2.hkl

Supporting information file. DOI: 10.1107/S2414314624011441/bt4161Isup3.cml

CCDC reference: 2405160

Additional supporting information:  crystallographic information; 3D view; checkCIF report

## Figures and Tables

**Table 1:** Hydrogen-bond geometry (Å, °)

*D*—H⋯*A*	*D*—H	H⋯*A*	*D*⋯*A*	*D*—H⋯*A*
O8—H8*O*⋯O7^i^	0.90 (2)	1.75 (2)	2.6547 (13)	178 (2)

Symmetry code: (i) 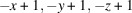.

**Table 2:** Experimental details

Crystal data	
Chemical formula	C_12_H_20_O_4_
*M*_r_	228.28
Crystal system, space group	Monoclinic, *C*2/*c*
Temperature (K)	120
*a*, *b*, *c* (Å)	9.8411 (4), 12.3319 (5), 21.0912 (10)
β (°)	94.254 (4)
*V* (Å^3^)	2552.56 (19)
*Z*	8
Radiation type	Mo *K*α
μ (mm^−1^)	0.09
Crystal size (mm)	0.55 × 0.29 × 0.25

Data collection	
Diffractometer	Stoe *IPDS* 2T
Absorption correction	Integration [*X-RED32* (Stoe & Cie, 2020 ▸), absorption correction by Gaussian integration (Coppens, 1970 ▸)]
*T*_min_, *T*_max_	0.966, 0.982
No. of measured, independent and observed [*I* > 2σ(*I*)] reflections	6713, 3019, 2607
*R*_int_	0.023
(sin θ/λ)_max_ (Å^−1^)	0.658

Refinement	
*R*[*F*^2^ > 2σ(*F*^2^)], *wR*(*F*^2^), *S*	0.040, 0.110, 1.03
No. of reflections	3019
No. of parameters	215
H-atom treatment	All H-atom parameters refined
Δρ_max_, Δρ_min_ (e Å^−3^)	0.35, −0.17

Computer programs: *X-AREA WinXpose*, *Recipe* and *Integrate* (Stoe & Cie, 2020 ▸), *SHELXT2014* (Sheldrick, 2015*a* ▸), *SHELXL2019/2* (Sheldrick, 2015*b* ▸) and *PLATON* (Spek, 2020 ▸).

## full crystallographic data

### rac-2-{3-[1-(Acetyloxy)ethyl]-2,2-dimethylcyclobutyl}acetic acid Crystal data

**Table:**

C_12_H_20_O_4_	*F*(000) = 992
*M**_r_* = 228.28	*D*_x_ = 1.188 Mg m^−^^3^
Monoclinic, *C*2/*c*	Mo *K*α radiation, λ = 0.71073 Å
*a* = 9.8411 (4) Å	Cell parameters from 9202 reflections
*b* = 12.3319 (5) Å	θ = 2.7–28.4°
*c* = 21.0912 (10) Å	µ = 0.09 mm^−^^1^
β = 94.254 (4)°	*T* = 120 K
*V* = 2552.56 (19) Å^3^	Block, colourless
*Z* = 8	0.55 × 0.29 × 0.25 mm

### rac-2-{3-[1-(Acetyloxy)ethyl]-2,2-dimethylcyclobutyl}acetic acid Data collection

**Table:**

Stoe IPDS 2T diffractometer	3019 independent reflections
Radiation source: sealed X-ray tube, 12x0.4mm long-fine focus	2607 reflections with *I* > 2σ(*I*)
Detector resolution: 6.67 pixels mm^-1^	*R*_int_ = 0.023
rotation method, ω scans	θ_max_ = 27.9°, θ_min_ = 2.7°
Absorption correction: integration [X-Red32 (Stoe & Cie, 2020), absorption correction by Gaussian integration (Coppens, 1970)]	*h* = −12→12
*T*_min_ = 0.966, *T*_max_ = 0.982	*k* = −16→16
6713 measured reflections	*l* = −27→22

### rac-2-{3-[1-(Acetyloxy)ethyl]-2,2-dimethylcyclobutyl}acetic acid Refinement

**Table:**

Refinement on *F*^2^	Primary atom site location: dual
Least-squares matrix: full	Hydrogen site location: difference Fourier map
*R*[*F*^2^ > 2σ(*F*^2^)] = 0.040	All H-atom parameters refined
*wR*(*F*^2^) = 0.110	*w* = 1/[σ^2^(*F*_o_^2^) + (0.0554*P*)^2^ + 1.746*P*] where *P* = (*F*_o_^2^ + 2*F*_c_^2^)/3
*S* = 1.03	(Δ/σ)_max_ < 0.001
3019 reflections	Δρ_max_ = 0.35 e Å^−^^3^
215 parameters	Δρ_min_ = −0.17 e Å^−^^3^
0 restraints	

### rac-2-{3-[1-(Acetyloxy)ethyl]-2,2-dimethylcyclobutyl}acetic acid Special details

**Table:**

Geometry. All esds (except the esd in the dihedral angle between two l.s. planes) are estimated using the full covariance matrix. The cell esds are taken into account individually in the estimation of esds in distances, angles and torsion angles; correlations between esds in cell parameters are only used when they are defined by crystal symmetry. An approximate (isotropic) treatment of cell esds is used for estimating esds involving l.s. planes.
Refinement. Hydrogen atoms were freely refined, constraining the displacement parameters of H atoms bonded to the same C atom to the same values.

### rac-2-{3-[1-(Acetyloxy)ethyl]-2,2-dimethylcyclobutyl}acetic acid Fractional atomic coordinates and isotropic or equivalent isotropic displacement parameters (Å^2^)

**Table:**

	*x*	*y*	*z*	*U*_iso_*/*U*_eq_	
C1	0.40274 (11)	0.82757 (9)	0.44466 (5)	0.0212 (2)	
H1	0.3386 (14)	0.8179 (12)	0.4752 (7)	0.021 (3)*	
C2	0.32858 (11)	0.80492 (9)	0.37750 (5)	0.0212 (2)	
C3	0.30462 (11)	0.93023 (9)	0.37194 (5)	0.0210 (2)	
H3	0.2131 (14)	0.9476 (11)	0.3853 (7)	0.022 (3)*	
C4	0.41585 (12)	0.94836 (9)	0.42633 (6)	0.0230 (2)	
H4A	0.3951 (15)	1.0022 (13)	0.4595 (8)	0.028 (3)*	
H4B	0.5044 (15)	0.9629 (12)	0.4101 (7)	0.028 (3)*	
C5	0.53464 (12)	0.76965 (10)	0.46563 (6)	0.0255 (3)	
H5A	0.6042 (16)	0.7761 (13)	0.4351 (8)	0.033 (3)*	
H5B	0.5742 (16)	0.8011 (13)	0.5037 (8)	0.033 (3)*	
C6	0.51864 (12)	0.65089 (9)	0.47940 (5)	0.0227 (2)	
O7	0.40910 (9)	0.60728 (7)	0.48459 (5)	0.0320 (2)	
O8	0.63537 (9)	0.59850 (8)	0.48631 (5)	0.0330 (2)	
H8O	0.619 (2)	0.5291 (17)	0.4968 (10)	0.052 (5)*	
C9	0.42329 (14)	0.76090 (11)	0.32965 (6)	0.0285 (3)	
H9A	0.4498 (16)	0.6864 (14)	0.3401 (8)	0.034 (2)*	
H9B	0.5078 (16)	0.8031 (13)	0.3281 (8)	0.034 (2)*	
H9C	0.3767 (16)	0.7584 (13)	0.2863 (8)	0.034 (2)*	
C10	0.19969 (13)	0.73714 (10)	0.37673 (6)	0.0269 (3)	
H10A	0.1379 (17)	0.7659 (14)	0.4077 (8)	0.039 (2)*	
H10B	0.2197 (16)	0.6612 (15)	0.3872 (8)	0.039 (2)*	
H10C	0.1509 (17)	0.7390 (14)	0.3334 (9)	0.039 (2)*	
C11	0.32282 (12)	0.98916 (10)	0.31042 (6)	0.0247 (3)	
H11	0.4032 (15)	0.9670 (12)	0.2925 (7)	0.024 (3)*	
C12	0.32133 (16)	1.11163 (11)	0.31801 (7)	0.0358 (3)	
H12A	0.3994 (18)	1.1323 (15)	0.3481 (9)	0.045 (3)*	
H12B	0.2328 (19)	1.1347 (15)	0.3341 (9)	0.045 (3)*	
H12C	0.3331 (17)	1.1457 (15)	0.2758 (9)	0.045 (3)*	
O13	0.20759 (8)	0.95683 (8)	0.26638 (4)	0.0268 (2)	
C14	0.22864 (12)	0.95332 (10)	0.20432 (6)	0.0250 (3)	
O15	0.33550 (9)	0.97409 (10)	0.18328 (5)	0.0382 (3)	
C16	0.10262 (15)	0.92018 (13)	0.16498 (7)	0.0345 (3)	
H16A	0.025 (3)	0.940 (2)	0.1833 (14)	0.099 (5)*	
H16B	0.102 (3)	0.847 (3)	0.1585 (14)	0.099 (5)*	
H16C	0.106 (3)	0.951 (2)	0.1231 (16)	0.099 (5)*	

### rac-2-{3-[1-(Acetyloxy)ethyl]-2,2-dimethylcyclobutyl}acetic acid Atomic displacement parameters (Å^2^)

**Table:**

	*U*^11^	*U*^22^	*U*^33^	*U*^12^	*U*^13^	*U*^23^
C1	0.0229 (5)	0.0191 (5)	0.0212 (5)	0.0011 (4)	−0.0002 (4)	0.0006 (4)
C2	0.0227 (5)	0.0198 (5)	0.0209 (5)	−0.0007 (4)	0.0006 (4)	−0.0001 (4)
C3	0.0212 (5)	0.0205 (5)	0.0211 (5)	0.0004 (4)	−0.0006 (4)	0.0008 (4)
C4	0.0266 (6)	0.0184 (5)	0.0232 (6)	0.0010 (4)	−0.0038 (4)	−0.0004 (4)
C5	0.0238 (6)	0.0213 (6)	0.0307 (6)	0.0011 (4)	−0.0030 (5)	0.0020 (5)
C6	0.0251 (5)	0.0216 (5)	0.0211 (5)	0.0034 (4)	−0.0003 (4)	0.0000 (4)
O7	0.0248 (4)	0.0223 (4)	0.0490 (6)	0.0033 (3)	0.0036 (4)	0.0072 (4)
O8	0.0240 (4)	0.0237 (5)	0.0513 (6)	0.0044 (3)	0.0029 (4)	0.0077 (4)
C9	0.0315 (6)	0.0269 (6)	0.0273 (6)	0.0014 (5)	0.0050 (5)	−0.0042 (5)
C10	0.0267 (6)	0.0252 (6)	0.0284 (6)	−0.0050 (5)	−0.0013 (5)	0.0001 (5)
C11	0.0209 (5)	0.0278 (6)	0.0246 (6)	−0.0020 (4)	−0.0034 (4)	0.0041 (4)
C12	0.0419 (8)	0.0264 (6)	0.0376 (8)	−0.0038 (6)	−0.0082 (6)	0.0099 (5)
O13	0.0204 (4)	0.0375 (5)	0.0221 (4)	−0.0027 (3)	−0.0011 (3)	0.0039 (3)
C14	0.0247 (5)	0.0256 (6)	0.0246 (6)	0.0044 (4)	0.0006 (4)	0.0040 (4)
O15	0.0265 (5)	0.0596 (7)	0.0290 (5)	0.0018 (4)	0.0048 (4)	0.0074 (4)
C16	0.0332 (7)	0.0422 (8)	0.0271 (7)	−0.0029 (6)	−0.0033 (5)	−0.0010 (6)

### rac-2-{3-[1-(Acetyloxy)ethyl]-2,2-dimethylcyclobutyl}acetic acid Geometric parameters (Å, º)

**Table:**

C1—C5	1.5183 (15)	C9—H9A	0.976 (17)
C1—C4	1.5467 (15)	C9—H9B	0.983 (16)
C1—C2	1.5697 (15)	C9—H9C	0.992 (17)
C1—H1	0.942 (14)	C10—H10A	0.991 (17)
C2—C10	1.5182 (16)	C10—H10B	0.979 (18)
C2—C9	1.5235 (16)	C10—H10C	1.000 (18)
C2—C3	1.5664 (15)	C11—O13	1.4669 (13)
C3—C11	1.5092 (16)	C11—C12	1.5189 (18)
C3—C4	1.5421 (15)	C11—H11	0.942 (15)
C3—H3	0.987 (14)	C12—H12A	0.993 (19)
C4—H4A	0.996 (16)	C12—H12B	0.999 (19)
C4—H4B	0.976 (15)	C12—H12C	1.000 (19)
C5—C6	1.5036 (16)	O13—C14	1.3410 (15)
C5—H5A	0.977 (17)	C14—O15	1.1990 (15)
C5—H5B	0.948 (17)	C14—C16	1.4970 (18)
C6—O7	1.2169 (15)	C16—H16A	0.91 (3)
C6—O8	1.3166 (14)	C16—H16B	0.91 (3)
O8—H8O	0.90 (2)	C16—H16C	0.96 (3)
			
C5—C1—C4	116.13 (10)	C2—C9—H9A	110.6 (10)
C5—C1—C2	120.62 (10)	C2—C9—H9B	113.2 (10)
C4—C1—C2	89.29 (8)	H9A—C9—H9B	107.1 (13)
C5—C1—H1	110.1 (8)	C2—C9—H9C	111.0 (9)
C4—C1—H1	111.4 (9)	H9A—C9—H9C	106.0 (13)
C2—C1—H1	107.7 (8)	H9B—C9—H9C	108.7 (13)
C10—C2—C9	110.39 (10)	C2—C10—H10A	110.4 (10)
C10—C2—C3	114.92 (9)	C2—C10—H10B	111.7 (10)
C9—C2—C3	113.45 (10)	H10A—C10—H10B	108.5 (14)
C10—C2—C1	116.09 (10)	C2—C10—H10C	110.0 (10)
C9—C2—C1	113.26 (10)	H10A—C10—H10C	108.5 (14)
C3—C2—C1	87.10 (8)	H10B—C10—H10C	107.7 (14)
C11—C3—C4	116.54 (10)	O13—C11—C3	106.14 (9)
C11—C3—C2	120.84 (10)	O13—C11—C12	108.90 (10)
C4—C3—C2	89.58 (8)	C3—C11—C12	112.67 (11)
C11—C3—H3	108.0 (8)	O13—C11—H11	107.5 (9)
C4—C3—H3	111.7 (8)	C3—C11—H11	111.2 (9)
C2—C3—H3	109.2 (8)	C12—C11—H11	110.2 (9)
C3—C4—C1	88.78 (8)	C11—C12—H12A	108.0 (11)
C3—C4—H4A	116.6 (9)	C11—C12—H12B	109.5 (11)
C1—C4—H4A	116.2 (9)	H12A—C12—H12B	111.3 (15)
C3—C4—H4B	111.7 (9)	C11—C12—H12C	108.8 (11)
C1—C4—H4B	111.0 (9)	H12A—C12—H12C	109.0 (15)
H4A—C4—H4B	111.0 (13)	H12B—C12—H12C	110.2 (15)
C6—C5—C1	114.51 (10)	C14—O13—C11	117.31 (9)
C6—C5—H5A	107.2 (9)	O15—C14—O13	123.89 (11)
C1—C5—H5A	113.3 (9)	O15—C14—C16	124.51 (12)
C6—C5—H5B	106.1 (10)	O13—C14—C16	111.60 (11)
C1—C5—H5B	110.0 (10)	C14—C16—H16A	112.2 (18)
H5A—C5—H5B	105.1 (13)	C14—C16—H16B	110.6 (18)
O7—C6—O8	123.00 (11)	H16A—C16—H16B	109 (2)
O7—C6—C5	123.65 (10)	C14—C16—H16C	108.8 (17)
O8—C6—C5	113.35 (10)	H16A—C16—H16C	112 (2)
C6—O8—H8O	108.9 (12)	H16B—C16—H16C	104 (2)
			
C5—C1—C2—C10	106.21 (12)	C5—C1—C4—C3	141.48 (11)
C4—C1—C2—C10	−133.49 (10)	C2—C1—C4—C3	17.33 (9)
C5—C1—C2—C9	−23.05 (15)	C4—C1—C5—C6	−176.63 (10)
C4—C1—C2—C9	97.25 (11)	C2—C1—C5—C6	−70.71 (15)
C5—C1—C2—C3	−137.38 (11)	C1—C5—C6—O7	−12.44 (18)
C4—C1—C2—C3	−17.08 (8)	C1—C5—C6—O8	168.52 (11)
C10—C2—C3—C11	−104.29 (12)	C4—C3—C11—O13	178.53 (9)
C9—C2—C3—C11	24.05 (14)	C2—C3—C11—O13	71.76 (13)
C1—C2—C3—C11	138.19 (10)	C4—C3—C11—C12	−62.37 (14)
C10—C2—C3—C4	134.65 (10)	C2—C3—C11—C12	−169.14 (10)
C9—C2—C3—C4	−97.01 (11)	C3—C11—O13—C14	−149.15 (10)
C1—C2—C3—C4	17.13 (9)	C12—C11—O13—C14	89.30 (13)
C11—C3—C4—C1	−142.07 (10)	C11—O13—C14—O15	0.61 (18)
C2—C3—C4—C1	−17.37 (9)	C11—O13—C14—C16	−179.50 (11)

### rac-2-{3-[1-(Acetyloxy)ethyl]-2,2-dimethylcyclobutyl}acetic acid Hydrogen-bond geometry (Å, º)

**Table:**

*D*—H···*A*	*D*—H	H···*A*	*D*···*A*	*D*—H···*A*
O8—H8*O*···O7^i^	0.90 (2)	1.75 (2)	2.6547 (13)	178 (2)

Symmetry code: (i) −*x*+1, −*y*+1, −*z*+1.
